# Enzymatic Spirulina Extract Enhances the Vasodilation in Aorta and Mesenteric Arteries of Aged Rats

**DOI:** 10.3390/md23100395

**Published:** 2025-10-08

**Authors:** Michal S. Majewski, Mercedes Klett-Mingo, Carlos M. Verdasco-Martín, Cristina Otero, Mercedes Ferrer

**Affiliations:** 1 Departamento de Fisiología, Facultad de Medicina, Universidad Autónoma de Madrid, 28029 Madrid, Spain; michal.majewski@uwm.edu.pl (M.S.M.);; 2 Department of Pharmacology and Toxicology, Faculty of Medicine, University of Warmia and Mazury, 10-082 Olsztyn, Poland; 3 Centro Nacional de Alimentación, AESAN, Ministerio de Consumo, 28220 Madrid, Spain; 4 Departamento de Biocatálisis, Instituto de Catálisis y Petroleoquímica, Consejo Superior de Investigaciones Científicas, 28049 Madrid, Spain

**Keywords:** aging, antioxidant, nitric oxide, aorta, reactive oxygen species, spirulina enzymatic extract

## Abstract

Aging, one of the main factors associated with cardiovascular diseases, induces vascular modifications through nitric oxide (NO) release and oxidative stress. Based on the antioxidant properties of the non-enzymatic spirulina extract (non-Enz-Spir-E) and that degrading enzymes enhances the extract bioactivity, the aim of this study was to analyze the in vitro effect of an Alcalase-assisted Enz-Spir-E on the vasodilator function of conduit and resistance arteries (which differently contribute to blood pressure regulation) in aging. Therefore, thoracic aorta (TA) and mesenteric arteries (MA) from male Sprague–Dawley rats (20–22 months-old) were divided into two groups: non-incubated vessels and vessels exposed to Enz-Spir-E (0.1% *w*/*v*) for 3 h. The vasodilation to acetylcholine (ACh), sodium nitroprusside (SNP, a NO donor), carbon-monoxide-releasing molecule (CORM), and cromakalim (a potassium channel opener), as well as NO and superoxide anion production, were studied. Enz-Spir-E increased the ACh-, SNP-, and CORM-induced responses in both types of arteries, while the cromalakim-induced relaxation was increased only in MA. Enz-Spir-E increased NO release (TA: 5.69-fold; MA: 1.79-fold), while it reduced superoxide anion formation (TA: 0.52-fold; MA: 0.66-fold). These results indicate that Enz-Spir-E improves aging-associated vasodilation through increasing NO release/bioavailability in both types of arteries and hyperpolarizing mechanisms only in MA.

## 1. Introduction

Cardiovascular diseases (CVD) are recognized as the primary cause of death globally, particularly among the elderly population [1]. Aging is a physiological process that leads to vascular dysfunction [2,3]. This dysfunction involves changes in both endothelial and smooth muscle cells, resulting in altered release/function of various endothelial factors, which contribute to vascular remodeling [2,3,4]. Among these endothelial factors, nitric oxide (NO), due to its vasodilatory and antiproliferative [5,6,7,8] actions, is a central molecule to regulating vasomotor tone. Variations in oxidative stress levels are also crucial for maintaining vascular function. During aging, an increase in reactive oxygen species (ROS) has been observed [9,10,11]. Excess ROS can affect a wide array of targets, causing modifications in proteins, lipids, and DNA structure, contributing to cellular senescence and death [12,13,14,15]. Moreover, ROS and other free radicals can trigger inflammatory responses, which, in turn, lead to the overproduction of additional radicals [16]. Of particular interest is the superoxide anion scavenging effect on NO, which reduces NO bioavailability [17,18].

Under pathophysiological conditions in which the ROS formation is enhanced, the nuclear factor erythroid 2-related factor 2 (Nrf2) is activated [16], which regulates different genes expression to protect cells against oxidative stress [19,20]. Among Nrf2 targets, hemeoxygenase-1 stands out. This enzyme synthesizes carbon monoxide (CO) and bilirubin, both of which possess antioxidant properties [21,22]. Furthermore, CO is also capable of inducing vasodilation by activating guanylate cyclase [23] and potassium channels [24]. In addition to these actions, the anti-apoptotic and anti-inflammatory effects of CO have also been documented [25,26].

The association between increased oxidative stress and age-induced vascular dysfunction has been reported [9,10,12] and, therefore, recommendations on antioxidant therapies have increased to ameliorate vascular dysfunction. As a result, numerous studies have explored various compounds with antioxidant properties [17,27,28,29,30,31]. Furthermore, there is increasing interest in the use of natural substances, such as microalgae extracts, for treating CVD. Among these, the filamentous cyanobacteria Arthrospira, particularly the species Arthrospira platensis and Arthrospira maxima (commercially known as Spirulina), have been recognized to possess antioxidant, antihypertensive, antidiabetic, and lipid-lowering properties [31,32,33,34]. Our research group has previously described some of the mechanisms behind the effects of non-enzymatic spirulina extracts in rat aorta from various vascular dysfunction models, including hypertension [35] and aging [36]. Moreover, it is well-established that the use of different degrading enzymes can improve extraction yields [37,38] and proteolytic digestion with Alcalase^®^ enzyme also enhances the bioactivity of the extract [39]. Thus, in that study, the Enz-Spir-E, obtained with Alcalase-assisted extraction, displayed superior bioactive properties compared to the rest of the extracts (using other proteases, cellulases, and lipases or without any enzyme biomass pretreatment). Considering that Enz-Spir-E greatly differs from the formerly studied non-enzymatic extract [35], the current study was proposed to investigate the effect of this extract in vessels of aged rats. Since large conduit and resistance arteries differently contribute to the total peripheral resistance, it is of great value to investigate the impact of the same Enz-Spir-E on the vasodilator responses of thoracic aorta and mesenteric arteries.

Therefore, the current investigation aims to examine the effect of the Enz-Spir-E on endothelium-dependent and endothelium-independent vasodilatory response induced by acetylcholine (ACh), sodium nitroprusside (SNP, a NO donor), carbon-monoxide-releasing molecules (CORM), and cromakalim (a potassium channel opener). Additionally, this study also explores the effects of the Enz-Spir-E on NO and superoxide anion levels.

## 2. Results

### 2.1. Composition and Antioxidant Activity of the Enz-Spir-E

The weight yield of the freeze-dried Enz-Spir-E obtained under optimal conditions [39] was determined with respect to the weight of dry biomass subjected to extraction (36.50 ± 0.10%).

Enz-Spir-E was free of toxic elements (0 ppm Hg, 0.1 ppm Cd, 0.7 ppm As, and 0.7 ppm Ni) and had variable amounts of other elements, including some essential elements (12737 ppm K, 1581 ppm Mg, 5.91 ppm Mn, 515 ppm Ca, 0.9 ppm Cu, 8.0 ppm Fe, 0 ppm Se, and 11 ppm Zn).

The total carbohydrate content of the Enz-Spir-E was 226 ± 13 mg/g extract.

This lyophilized Enz-Spir-E had a relatively high amino acid content (45% *w*/*w* lyophilized extract), containing all types of amino acids, particularly the essential ones (Table 1).

According to the proteomic analyses, the Enz-Spir-E contains eight peptides (Table 2) produced from different proteins of Arthrospira sp. and their corresponding biological activities: MKKIEAIIRPF (nitrogen regulatory protein P–II [Arthrospira platensis NIES–39]); ALAVGIGSIGPGLGQGQQ (AtpH [Arthrospira platensis HN01]); TTAASVIAAA (ATP synthase c chain [Arthrospira platensis NIES–39]); and DFPGDDIPIVS (full = elongation factor Tu; short = EF-Tu). Several of these peptides were fragments of ATP synthase.

The Enz-Spir-E had also a polyphenol content determined by the Folin–Ciocalteu method (FCR) of 11.21 ± 0.02 mg/g extract and antioxidant activities determined by the ABTS and ORAC methods of 19.7 ± 0.5 and 462 ± 19 TEAC (µmol Trolox/g extract), respectively.

### 2.2. Animal Weight and Systolic Blood Pressure

The animal weight (709.4 ± 10.9 g) and systolic blood pressure of the aged rats (162 ± 4.7 mmHg) was the same as that reported in a previous study [35] and in line with earlier investigations [40].

### 2.3. Effect of Enz-Spir-E on Vascular Functioning

The vascular contraction induced by 75 mM KCl was similar within each artery type. In aorta, control: 2162.0 ± 68.7 mg; Enz-Spir-E: 2094.0 ± 111.9 (*p* > 0.05). In mesenteric artery, control: 1190.0 ± 188.7; Enz-Spir-E: 1327.0 ± 108.7 (*p* > 0.05).

**Figure 1:**
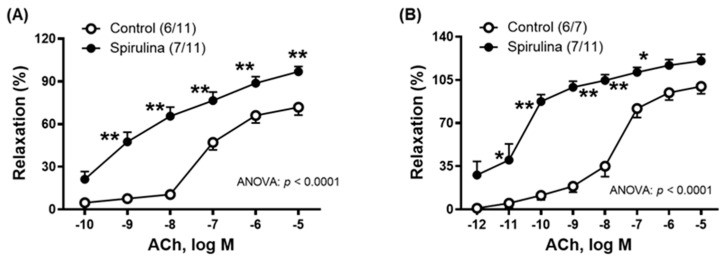

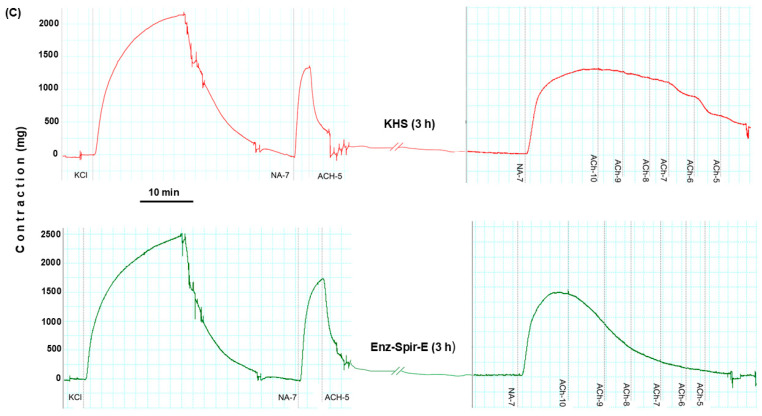
Effect of the Enz-Spir-E (0.1% *w*/*v*) on the vasodilator response induced by acetylcholine (ACh). Effect of the spirulina enzymatic extract incubation on the concentration–response curve to Ach in aortic (**A**) and mesenteric (**B**) arterial segments from aged SD rats. Results (means ± SEM) are represented as the percentage of inhibition of the contraction elicited by 0.1 µM noradrenaline (aorta: 67.4 ± 3.1% in control and 60.9 ± 5.6% in spirulina group; *p* > 0.05) and 1 µM noradrenaline (mesenteric arteries: 70.8 ± 4.3% in control and 93.1 ± 5.6% in spirulina group; *p* < 0.05). Number of animals and arterial segments are indicated in parentheses. The statistical significances are indicated in the corresponding graphs. * *p* < 0.05; ** *p* < 0.01 compared to control condition. (**C**) Representative portions of the original recording of aortic segments showing the effect of Enz-Spir-E on the concentration–response curve to ACh. Note that Enz-Spir-E incubation was initiated after checking vascular functional integrity (through KCl-induced response) and endothelium functionality (through ACh-induced vasodilation in NA-precontracted segments). Individual experimental data points are included in the Supplementary Materials.

The incubation of vessels with the Enz-Spir-E (0.1% *w*/*v*) produced an increase in the vasodilator response to ACh (0.1 nM–10 µM) in both aorta and mesenteric artery (Figure 1A,B). As indicated in the portion of the original recording of aortic segments, the Enz-Spir-E was added after checking vascular functional integrity (through KCl response) and endothelium functionality (through ACh-induced vasodilation in NA-precontracted segments) (Figure 1C). 

**Figure 2:**
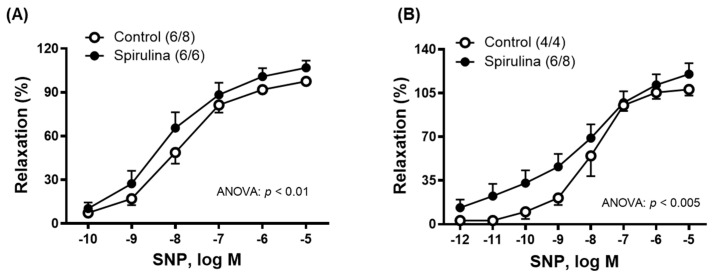
Effect of the Enz-Spir-E (0.1% *w*/*v*) on the vasodilator response induced by sodium nitroprusside (SNP). Effect of the spirulina enzymatic extract incubation on the concentration–response curve to SNP in aortic (**A**) and mesenteric (**B**) arterial segments from aged SD rats. Results (means ± SEM) are represented as the percentage of inhibition of the contraction elicited by 0.1 µM noradrenaline in aorta (51.7 ± 3.1% in control and 71.2 ± 14.9% in spirulina group; *p* < 0.05) and 1 µM noradrenaline in mesenteric arteries (70.8 ± 4.7% in control and 71.2 ± 14.9% in spirulina group; *p* < 0.05). Number of animals and arterial segments are indicated in parentheses. The statistical significances are indicated in the corresponding graphs.

Since ACh can induce the release of different endothelial factors, such as NO or CO, among others, the possible effect of Enz-Spir-E on the sensitivity of smooth muscle cells to NO or CO and the vasodilator response induced by NO and CO donors, sodium nitroprusside (SNP) or carbon-monoxide-releasing molecule (CORM), respectively, were studied. After incubation with the Enz-Spir-E, the vasodilator response induced by SNP (0.1 nM–10 µM) increased more in mesenteric arteries (Figure 2B) than in the aorta (Figure 2A).

**Figure 3:**
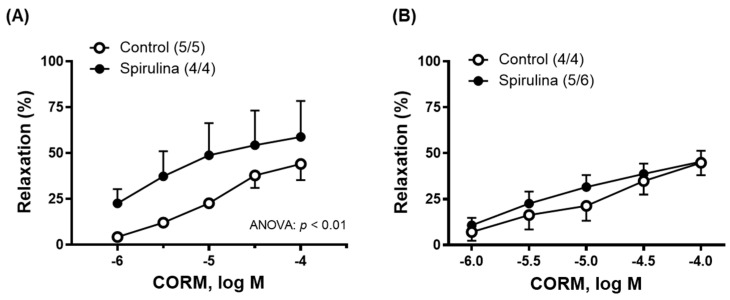
Effect of the Enz-Spir-E (0.1% *w*/*v*) on the vasodilator response induced by CO-releasing molecule (CORM). Effect of the spirulina enzymatic extract incubation on the concentration–response curve to CORM in aortic (**A**) and mesenteric (**B**) arterial segments from aged SD rats. Results (means ± SEM) are represented as the percentage of inhibition of the contraction elicited by 0.1 µM noradrenaline in aorta (45.0 ± 51% in control and 62.8 ± 6.7% in spirulina group; *p* > 0.05) and 1 µM noradrenaline in mesenteric arteries (96.7 ± 3.6% in control and 85.2 ± 6.4% in spirulina group; *p* > 0.05). Number of animals is indicated in parentheses. The statistical significances are indicated in the corresponding graphs.

The vasodilator response induced by CORM (1 µM–100 µM) increased after vessels were incubated with the Enz-Spir-E, especially in the aortic segments (Figure 3A,B).

**Figure 4:**
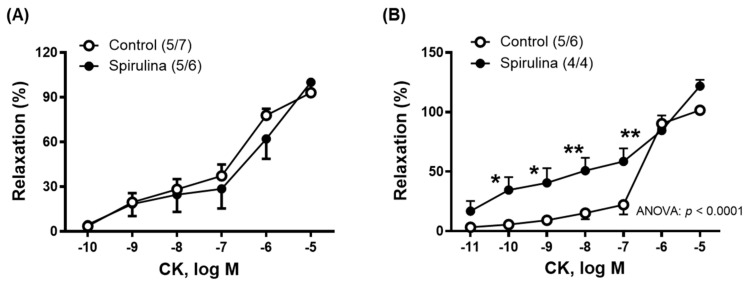
Effect of the Enz-Spir-E (0.1% *w*/*v*) on the vasodilator response induced by the K_ATP_ channel opener cromakalim (CK). Effect of the spirulina enzymatic extract incubation on the concentration–response curve to CK in aortic (**A**) and mesenteric (**B**) arterial segments from aged SD rats. Results (means ± SEM) are represented as the percentage of inhibition of the contraction elicited by 0.1 µM noradrenaline in aorta (50.7 ± 3.4% in control and 51.7 ± 4.6% in spirulina group; *p* > 0.05) and 1 µM noradrenaline in mesenteric arteries (93.8 ± 4.2% in control and 80.2 ± 5.2% in spirulina group; *p* > 0.05). Number of animals and arterial segments are indicated in parentheses. The statistical significances are indicated in the corresponding graphs. * *p* < 0.05; ** *p* < 0.01 compared to control condition.

To investigate the potential effect of the Enz-Spir-E on the function of potassium channels, a concentration–response curve of the K_ATP_ channel opening agent cromakalim (CK, 0.1 nM–10 µM) was generated (Figure 4). Increased vasodilator response to CK after Enz-Spir-E incubation was found, particularly in mesenteric arterial segments (Figure 4B).

Values of EC_50_, E_max_, and AUC for the vasodilator responses are included in Table 3.

### 2.4. Effect of Enz-Spir-E on Nitric Oxide Release

**Figure 5:**
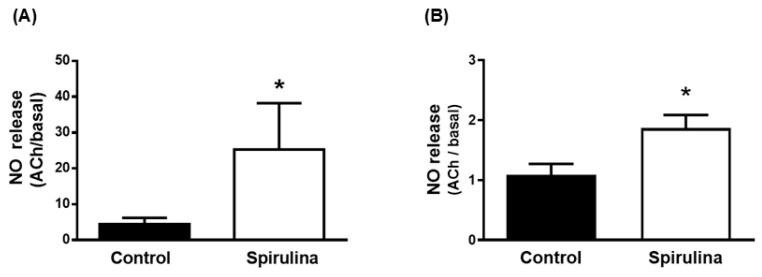
Effect of the Enz-Spir-E (0.1% *w*/*v*) on NO release. Effect of the spirulina enzymatic extract incubation on the acetylcholine-induced NO release in aortic (**A**) and mesenteric (**B**) arterial segments from aged SD rats. Results (means ± SEM) are represented as the ratio between the ACh- and basal-induced NO release. Number of animals: 4. * *p* < 0.05 compared with the control condition.

In the aortic segments, stimulation with ACh increased the emitted fluorescence more in the arteries incubated with the Enz-Spir-E than in the control arteries (Figure 5A). In mesenteric arteries, ACh also increased nitrite release more in arteries incubated with the Enz-Spir-E than in control arteries (Figure 5B). Note that the effect of Enz-Spir-E was more pronounced in the aorta than in the mesenteric artery: 5.69-fold in aorta (control: 4.4 ± 1.8; spirulina: 25.3 ± 12.9; *p* < 0.05) and 1.79-fold in mesenteric artery (control: 1.1 ± 0.2; spirulina: 1.87 ± 0.20; *p* < 0.05).

### 2.5. Effect of Enz-Spir-E on Superoxide Anion Production

**Figure 6:**
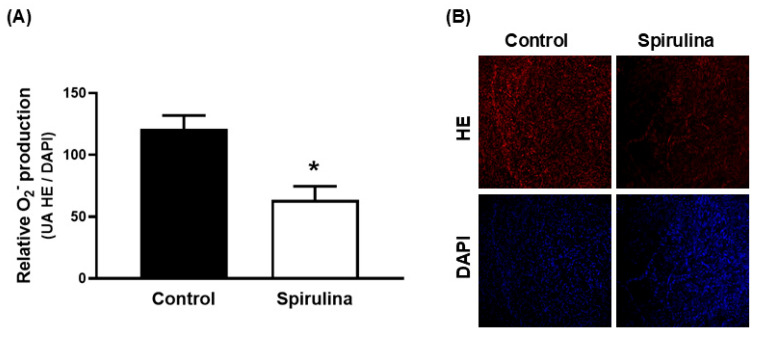
Effect of the Enz-Spir-E (0.1% *w*/*v*) on the production of superoxide anion in aorta. Effect of the spirulina enzymatic extract incubation on the production of superoxide anion in aortic segments from aged SD rats. Quantitative analysis (**A**) and representative confocal images (**B**) showing in situ detection of superoxide anion in red and DAPI-nuclei staining in blue. Results (means ± SEM) are expressed as the ratio between the fluorescence emitted by HE and that emitted by DAPI. Number of animals: 4. * *p* < 0.05 compared with control condition.

In aortic segments from aged SD rats, Enz-Spir-E incubation significantly reduced the production of superoxide anion, as indicated by diminished HE fluorescence (Figure 6).

**Figure 7:**
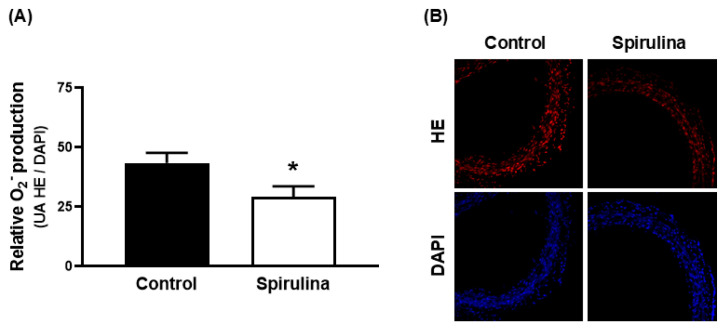
Effect of the Enz-Spir-E (0.1% *w*/*v*) on the production of superoxide anion in mesenteric artery. Effect of the spirulina enzymatic extract incubation on the production of superoxide anion in mesenteric arterial segments from aged SD rats. Quantitative analysis (**A**) and representative confocal images (**B**) showing in situ detection of superoxide anion in red and DAPI-nuclei staining in blue. Results (means ± SEM) are expressed as the ratio between the fluorescence emitted by HE and that emitted by DAPI. Number of animals: 3. * *p* < 0.05 compared with control condition.

Similar results were found in mesenteric arterial segments, in which incubation with the Enz-Spir-E significantly reduced the production of superoxide anion with respect to the control condition (Figure 7). Superoxide anion formation was reduced to a similar extent in aortic or in mesenteric artery segments: 0.52-fold in aorta (control: 93.2 ± 7.5; spirulina: 65.6 ± 3.6; *p* < 0.05) and 1.79-fold in mesenteric artery (control: 43.5 ± 4.2; spirulina: 29.0 ± 4.5; *p* < 0.05).

## 3. Discussion

The Enz-Spir-E significantly differs in composition and bioactivities with respect to the non-enzymatic extract [37,39]. The Enz-Spir-E had a specific set of peptides (Table 2) and more bioactive components (higher antioxidant, polyphenol, and amino acid contents, Table 1) than the non-enzymatic extract. Polyphenol content is 2.3 times higher than that of the non-enzymatic extract, having 7.3 times more antihypertensive activity and 4.4 times and 1.5 times higher antioxidant activities (ORAC and ABTS, respectively) than the non-enzymatic extract [37,39]. These differences prompted us to investigate the effect of Enz-Spir-E on the vasodilation in aorta and mesenteric arteries of aged rats.

The current study demonstrated that incubation with the Enz-Spir-E enhanced vasodilatory function in both conduit and resistance arteries of aged rats. A 3 h exposure to the enzymatic extract led to a reduction in oxidative stress in both vessel types, which may have facilitated an increased NO release/function. However, the mechanisms underlying the increased vasodilator function induced by the Enz-Spir-E appear to differ between the aorta and the mesenteric artery.

The aging process negatively impacts various biochemical and physiological parameters [12,14,16,41]. A key characteristic of aging is oxidative stress, characterized by an imbalance in favor of pro-oxidant over antioxidant systems [42]. Excessive ROS production leads to cellular and tissue damage, contributing to functional decline [43]. In the vascular system, elevated oxidative stress reduces NO bioavailability and function, thereby promoting alterations in the vascular function and structure [2,3,4,9,44]. Consequently, numerous intervention strategies have been developed to target oxidative stress, often involving compounds with antioxidant properties [17,27,28,29,30,31,45]. Among these, natural substances, particularly microalgae-derived extracts such as those from spirulina, have garnered growing interest due to their demonstrated antioxidant, antihypertensive, and antidiabetic effects [32,33,34]. The Enz-Spir-E employed in this study exhibits significant antioxidant activity, probably due to its important polyphenol content. Notably, the antioxidant capacity of the Enz-Spir-E, as assessed by the ORAC method, was substantially higher than that observed with a non-enzymatic aqueous extract previously reported by our research group [37,39]. The data also confirmed the antioxidant effects of the Enz-Spir-E on both the aorta and mesenteric artery, evidenced by a reduction in superoxide anion levels within the vascular wall. These findings are consistent with previous observations using a non-enzymatic spirulina extract in arteries exhibiting vascular dysfunction in both hypertensive [35] and aging [36] conditions. Given the role of superoxide anion in NO elimination, the impact of Enz-Spir-E on NO levels was also examined. Incubation with the Enz-Spir-E was found to enhance ACh-induced NO release, corroborating earlier studies [35,36,46] and also in line with studies describing increased endothelium-dependent relaxation by compounds that reduce oxidative stress. Furthermore, this effect was more pronounced in the aorta than in the mesenteric artery, suggesting that the Enz-Spir-E could differently modulate eNOS activation and/or potential differences in the NO contribution to vascular tone regulation depending on the vessel type. These aspects will be further addressed in the context of spirulina influence on vasodilatory responses.

To achieve this goal, endothelium-dependent relaxation after Enz-Spir-E incubation was first evaluated. The findings revealed that ACh-induced relaxation was augmented in both the thoracic aorta and the mesenteric artery following Enz-Spir-E treatment. These results are consistent with earlier studies performed in arteries of hypertensive [35] and aged [36] rat treated with a non-enzymatic Spir-E. It is widely recognized that, apart from NO, other vasodilatory mediators contribute to the ACh-induced response, and their relative involvement can vary depending on the vascular bed [47,48]. In particular, numerous studies have highlighted the significance of hyperpolarizing mechanisms, predominantly in resistance arteries rather than conduit vessels, though this also depends on the physiological condition of the vessel. In this regard, our research group has previously demonstrated an increase in hyperpolarization in aortic segments associated with diminished NO bioavailability due to elevated superoxide levels [29].

Because aging impairs the efficacy of the NO–cGMP–PKG signaling cascade [4,49,50], the effect of Enz-Spir-E on vascular smooth muscle responsiveness to NO was further analyzed. The data revealed that the relaxation induced by SNP was enhanced in both the aorta and mesenteric artery following Enz-Spir-E incubation, corroborating prior reports from various research teams [35,36,46].

Since CO can also promote vasorelaxation and may contribute to ACh-mediated responses, the influence of Enz-Spir-E on CO-induced relaxation was assessed. Results showed that CORM-induced vasorelaxation increased in aortic segments but remained unchanged in mesenteric arteries. Taking into account that NO and CO induce similar mechanisms of action —both capable of activating guanylate cyclase [23] and potassium channels [24] —and considering that the function of K_ATP_ channels has been reported to decline with aging [48,51], the potential effect of the Enz-Spir-E on K_ATP_ channel function was assessed. To carry out this goal, the vasodilatory response elicited by cromakalim, a K_ATP_ channel opener, was found to be enhanced by the Enz-Spir-E exclusively in the mesenteric artery. This striking result could be explained by the differential expression of K_ATP_ channel isoforms according to the vessel types [52]. In addition, it is as if the response to cromakalim had two vasorelaxant components, one with high affinity (at concentrations up to 0.1 µM) and the other with low affinity (at concentrations greater than 0.1 µM). It appears that the Enz-Spir-E, only in mesenteric arteries, would be able to increase the affinity/sensitivity to cromakalim at low concentrations, although the EC_50_ values for the whole curve were not statistically different. These observations open a series of future studies in which the interactions between natural compounds and potassium channels would be worth analyzing.

Collectively, the findings regarding vasodilation across both vascular beds seem to indicate that the Enz-Spir-E facilitates hyperpolarizing mechanisms in the mesenteric artery, though these mechanisms appear to differ from those operating in the aorta. In this regard, it is important to point out that the ACh-, SNP-, and CK-induced relaxation exceeds 100% only in mesenteric artery, which could suggest an increased participation of hyperpolarizing mechanisms. Also, differences between aorta and mesenteric artery in the increased NO levels by the Enz-Spir-E, with a similar decrease in superoxide levels, could suggest specific actions on different targets depending on the type of artery. Furthermore, it is noteworthy that, in the present study, the vasodilator response to SNP in the aorta following incubation with the Enz-Spir-E was markedly lower than that observed with the non-enzymatic extract [36]. Similarly, cromakalim-induced relaxation was unaffected by the Enz-Spir-E, contrasting with previous findings using the non-enzymatic preparation. These differences highlight an emerging area of investigation, suggesting that the extraction method may influence the specific mechanisms of action in particular vascular tissues. Consequently, the choice of extractive technique should be carefully considered when evaluating the vascular effects of bioactive compounds.

Because of the rising rate of aging and the associated increase in mortality from age-related diseases, research into the mechanisms of action of various natural compounds has become increasingly relevant. This study contributes to that effort by demonstrating vessel-specific effects of an Enz-Spir-E. Notably, the enhancement of vasodilatory function in the mesenteric artery is particularly significant, as this vascular bed plays a critical role in blood pressure regulation. Further in vitro and in vivo studies are warranted to elucidate the vascular effects of different spirulina extracts and their mechanisms of action.

## 4. Materials and Methods

### 4.1. Animal Ethical Procedures

Sprague–Dawley (SD) rat is one of the most common rat strains often used in aging studies, and our research group has worked on it in different investigations [36,40,48]. Male SD rats, 20–22 months old (709.4 ± 10.9 g), were provided by the Animal Facility of the Universidad Autónoma de Madrid (UAM) (Establishment License number ES-28079-0000097). Animals were exposed to constant temperature, 12 h dark/light cycle, and standard feeding with rat chow and water ad libitum. Systolic blood pressure was indirectly measured in awake animals by the tail-cuff method (Letica, Digital Pressure Meter, LE5000, Barcelona, Spain), and the animals were weighed before sacrifice. Rats were sacrificed by CO_2_ inhalation and subsequent decapitation; the aorta and mesenteric arteries were carefully dissected and placed in Krebs–Henseleit solution (KHS) at 4 °C containing 115 mM NaCl, 2.5 mM CaCl_2_, 4.6 mM KCl, 1.2 mM KH_2_PO_4_, 1.2 mM MgSO_4_, 25 mM NaHCO_3_, and 11.1 mM glucose. The blood vessels were cleaned of adhering adipose and connective tissues, cut into rings 4 mm in length, and divided into two groups: a control group (arteries in the absence of Enz-Spir-E) incubated in KHS and a spirulina group (arteries exposed for 3 h to the Enz-Spir-E 0.1% *w*/*v*). All animal protocols were approved by the Research Ethics Committee of UAM according to directives 63/2010/UE and R.D. 53/2013 of the Ministerio de Agricultura, Pesca y Alimentación of Spain (Project License number: PROEX 182.7/21). The experiments were conducted in accordance with the published Guiding Principles in the Care and Use of Animals approved by the European Union directives 63/2010 and ARRIVE guidelines and Spanish regulation RD53/2013.

### 4.2. Enzymatic Spirulina Extract

#### 4.2.1. Enzymatic-Assisted Extraction

The biomass used for extraction was a dry powder of *Arthrospira platensis* from ASN Leader S.L. (Murcia, Spain). The biomass (spirulina) was for nutritional use with the following composition: 50–65% proteins, 6–7.5% lipids, 18–22% carbohydrates, 15% minerals, 0.2% fiber, and 390 cal/100 g (according to the manufacturer, unknown analytical methods). The biomass was first degraded by Alcalase^®^ 2.4 L FG (commercial enzyme preparation, kindly donated by Novozymes A/S, Denmark) and then its biocomponents were extracted and dehydrated. The proteolytic enzyme had 2.4 AU-A/g activity units, determined by the manufacturer by the kinetic dimethyl casein method. The method is based on the hydrolysis of dimethyl casein (DMC) by Alcalase^®^ to small peptides. As soon as the primary amino groups are formed, trinitrobenzene sulfonic acid (TNBS) forms a colored complex with them. The absorption change at 420 nm per time unit was calculated. A <Cobas> FARA centrifugal analyzer from Roche was employed. The reaction was carried out at 50 °C and pH 8.3 for 9 min, using different enzyme concentrations in the range, 0.072–0.216 mAU(A)/mL. Measuring time was 3 min for 0.5–1.0 g of sample. Samples were diluted in a measuring flask with 2% sodium sulfite solution, then stirred for 15 min. on the magnetic stirrer and further diluted with 2% sodium sulfite solution using the diluter. The activity in the final dilution lies in the enzyme concentration range between standard 2 and 5 (0.090–0.180 mAU (A)/mL). Each weighing was analyzed once. Calculation of activity of a sample in AU(A)/g is performed as stated in the formula:

Activity AU(A)/g = S V F/W 1000

where S = reading from <Cobas>; FARA in mU/mL (=mAU(A)/mL); V = volume of the measuring flask used in ml; F = dilution factor for second dilution; W = weight of sample in g; and 1000 = conversion factor from mAU(A) to AU(A).

Alcalase^®^ is a commercial preparation of subtilsine A, a serine endopeptidase (EC. 3.4.21.62). It hydrolyzes amino esters as heterocyclic amino esters [53]. Conditions for biomass degradation were previously determined in a former study: 1% *v*/*w* Alcalase^®^ at pH 6.5 and 30 °C for 24 h [39]. Once the biomass suspension was digested, a solvent extraction of the biocomponents using a hexane–isopropanol mixture (3:2, *v*/*v*) was carried out. The obtained aqueous and oil phases were separated. The aqueous phase was lyophilized for 4 days and kept at −20 °C until used for this study.

#### 4.2.2. Elemental Analysis

The dry Enz-Spir-E (70–80 mg) was digested in Teflon glass with 6 mL HNO_3_ for 20 min in a Multiwave 3000 microwave ANTON PAAR model, and the program conditions were as follows: starting from 0 to 500 W for 5 min, maintaining at 500 W for 10 min, increasing to 1000 W for 10 min, and maintaining at 1000 W for 20 min, 240 °C maximal temperature program, and 60 Bar maximal pressure. After digestion, the acid solution was diluted to 25 mL, and a 0.5 mL aliquot of the resultant solution was again diluted to 10 mL for semiquantitative analysis. The samples were analyzed by inductively coupled plasma–mass spectrometry (ICP–MS) in a NexION 300XX apparatus from Perkin-Elmer using a method previously described [54]. The apparatus conditions were ICP RF power of 1100 W; 0.80 L/min nebulizer gas; flow-enabled Autolens; detector mode in dual mode; sample introduction system Ryton cross-flow with Scott spray chamber; nickel sampler/skimmer cones; 45 s at 48 rpm rinse time; 20 s at 48 rpm sample uptake time; 30 s at 20 rpm read delay; peak hopping for scanning mode; 25 ms per point dwell time; 20 sweeps/reading; and 2.01 min sample time. The blanks and standards used for the response curve were diluted in HNO_3_ 1% (*v*/*v*) using Milli Q water. The internal standard Rh had a final concentration of 30 μg/L. A response curve was built with heuristic calculations interpreting the mass spectra and correlating the intensity read for each ion at a known element concentration.

#### 4.2.3. Total Carbohydrate Content

The total carbohydrate content of the dried Enz-Spir-E was determined by the phenol–sulfuric method [55]. An extract solution in Milli–Q water (0.2 g/L) was analyzed in triplicate. The results are given as the calculated mean value with the standard deviation.

#### 4.2.4. Amino Acid Composition

The amino acids content was quantitatively determined using the method described by More [56]. An amino acid analyzer Biochrom 30 Series was used, with >0.5 CV reproducibility at 10 nmol. Qualitative and quantitative analyses (10 pmol sensitivity) were carried out after separation by ion-exchange liquid chromatography and a continuous postcolumn reaction with ninhydrin. Three replicas of the Enz-Spir-E (1 mg/mL), containing a known concentration of norleucine (internal standard), were prepared.

#### 4.2.5. Proteomic Analysis

Peptide identification of the Enz-Spir-E was carried out by liquid chromatography coupled to an electrospray ionization mass spectrometer in positive ionization mode (LC– ESI–MS/MS) to identify the biocomponents. The sample was cleaned with C18 tips, model ZipTip Pipette Tips C18 (ref. ZTC18S096 of Millipore), before the analysis. An Ultimate 3000 nanoHPLC (Dionex, Sunnyvale, CA, USA) coupled to an ion trap mass spectrometer (Amaz Speed, Bruker Daltonics, Bremen, Germany) was used. The reversed-phase analytic column used was an Acclaim C18 PepMap with 75 µm × 15 cm, 3 µm particle size and 100 Å pore size (ThermoScientific, Waltham, MA, USA). A C18 PepMap trap column (5 µm particle diameter and 100 Å pore size) was connected in series with the analytical column. The mobile phase was 0.1% trifluoroacetic acid in 98% water/2% acetonitrile solution (ScharLab, Barcelona, Spain) at 3 µL/min. The elution was performed with water (phase A) and 0.1% formic acid in 80% acetonitrile/20% water (phase B), containing 0.1% formic acid (Fluka, Buchs, Switzerland) at 300 nL/min and the following gradient: isocratic mode with 96% A/4% B for 5 min, a linear increase to 40% B in 60 min, a linear increase to 95% B in 1 min, and isocratic conditions of 95% B for 7 min, returning to initial conditions in 10 min. The extract solution (5 µL of 4 µg/µL solution) was injected and analyzed at 214 and 280 nm. For the second analysis, 5 µL of the extract solution (10 µg/µL) was analyzed. The LC apparatus with the ion trap spectrometer by a CaptiveSpray source (Bruker Daltonics, Bremen, Germany) operated in positive mode with a capillary voltage of 1400 V. Sequential acquisition of both MS spectra (*m*/*z* 350–1500) and MS CID spectra of the 8 most abundant ions was conducted. In the analyses of the 10 µg/µL samples, the MS spectral range was 100–1000 *m*/*z*. Exclusion dynamics were applied to prevent the isolation of the same *m*/*z* for 1 min after fragmentation.

For peptide identification, MS and MS/MS data were processed using Data Analysis 4.1 (Bruker Daltonics, Bremen, Germany). MS/MS spectra (generic Mascot files) were analyzed using a database from National Center for Biotechnology Information (NCBInr) and Mascot v.2.6.0 (Matrix Science, London, UK) [57] was employed. Oxidized methionine was used as the modification variable without enzyme restriction. The peptide mass tolerances were 0.3 Da and 0.4 Da in MS and MS/MS modes, respectively. Precision was ≤±0.1–0.2 Da for both the MS and MS/MS spectra. All MS and MS/MS spectra of the Alcalase^®^ extract were also analyzed with “de novo” tool of the Peaks software (Bioinformatics Solutions, Inc.), which combines an unconditioned “de novo” analysis of MS/MS spectra with the more conventional search against organism-specific (i.e., Arthrospira) sequence databases. The results table only includes MS and MS/MS spectra and their corresponding “de novo” interpretations. Sequences with confidence values equal to or greater than 80 were only considered. The following identities are not distinguishable: I and L; K and Q; and F and M (ox).

#### 4.2.6. Total Polyphenol Content

Polyphenols and other antioxidants content of Enz-Spir-E was determined by the Folin–Ciocalteu reagent (FCR) method [58]. The samples were prepared in 95% (*v*/*v*) methanol/water (10 g/L) and then sonicated (20 kHz) for 10 min. The results are given as the mean value obtained for three replicates with the corresponding standard deviation.

#### 4.2.7. Antioxidant Activity

The antioxidant activity of the Enz-Spir-E was determined by two different methods:

ABTS assay: A method for quantification of the ability of antioxidants of Enz-Spir-E to sequester a stable cation radical [59]. The preformed monocation radical of 2,29-azinobis-(3-ethylbenzothiazoline-6-sulphonic acid) (ABTS•+) is generated by the oxidation of ABTS with potassium persulphate, being reduced in the presence of a hydrogen donor antioxidant.

Hydroxyl radical scavenging assay (ORAC fluorescein assay): The method determined the hydroxyl radicals (OH●) in living organisms that participate in inflammation processes in damaged tissues related to oxidative stress. Dávalos et al.’s protocol was used [60].

Trolox standard solutions (40–200 µmol/L) in ethanol were used for calibration in the ABTS assay. A blank without the antioxidant solution and 10 calibration solutions using Trolox (10–100 μM final concentration) as antioxidant were also carried out in each ORAC assay.

For ABTS and ORAC analyses, a SynergyTM HT multimodal plate reader with automatic dispenser of samples and temperature control from Biotek Instruments (Winooski, VT, USA) was used. The software was Biotek Gen5TM. Each 96-well plate was analyzed in quadruplicate, with four standard levels of calibration and eight repetitions for the blank or control. The reaction started after the automatic addition of 60 μL of ABTS radical or AAPH to the sample solution for the ABTS and ORAC assays, respectively. The ABTS radical activity was determined after 10 min of reaction. The value was read after 180 min of reaction in the case of ORAC method. Activities were determined in quadruplicate (ABTS) and in triplicate (ORAC). The results are expressed as the Trolox equivalent antioxidant capacity (TEAC) in mmol Trolox/g extract as the concentration (mM) of a standard reference solution (Trolox) with an antioxidant capacity equivalent to that of a solution (1 mM) of the investigated analyte.

### 4.3. Vascular Function

This was previously described in detail [36,61]. Aortic rings were mounted in an organ bath filled with 5 mL of KHS at 37 °C and continuously aerated with 95% O_2_ and 5% CO_2_ mixture (pH = 7.4). The signal was processed with a force transducer (Grass FTO3C; Grass Instruments Co., Quincy, MA, USA) connected to a model 7D Grass polygraph. The aortic rings were subjected to a tension of 1 g and 0.5 g for mesenteric rings (according to the different arterial diameters) as in previous studies [29,40]. After the equilibration period of 90 min, the vessels were exposed to 75 mM KCl to assess functional integrity. Arterial segments that did not reach a KCl-induced contraction of 600 mg for mesenteric or 1000 mg for aortic segments were discarded. The integrity of vascular endothelium was checked with vasoconstrictor NA (0.1 µM aortic and 1 µM mesenteric rings) and dilated with ACh (10 µM). Only arterial rings in which ACh-induced vasodilation was greater than 60% were used.

Separate aortic and mesenteric rings were incubated with the Enz-Spir-E (0.1% *w*/*v*) for 3 h, changing with a fresh Enz-Spir-E every 60 min and cumulative concentration-response curves were constructed for ACh (0.1 nM–10 µM), SNP (the NO donor, 0.1 nM–10 µM), CORM (the CO donor, 1 µM–100 µM), and CK (the K_ATP_ channel opener 0.1 nM–10 µM) in NA precontracted rings. All concentration–response curves were constructed in the presence of the Enz-Spir-E.

### 4.4. Release of Nitric Oxide

The release of NO in mesenteric segments was measured by using a nitrite colorimetric assay kit (Cayman Chemical, 780001), as described previously [29,36]. During the last 30 min of the incubation period with the Enz-Spir-E, the last incubation media (changed every 10 min) was collected to measure the basal NO release. Both NA (1 µM, for 2 min) and ACh (10 µM, for 8 min) were added to measure the stimulated NO release. The analysis was carried out according to the manufacturer’s protocol. The data are expressed as the ratio between ACh-induced and basal nitrite release.

The release of NO in aortic segments was measured using the specific fluorescent probe 4,5-diaminofluorescein (DAF-2, Sigma-Aldrich D-225, St. Gallen, Switzerland). After the stabilization period and during the last 45 min of incubation with the Enz-Spir-E or KHS media, the arterial segments were transferred to a bath containing 0.5 μM DAF-2 for 45 min, as previously described [62]. Once the 500 μL baths were refilled, each with its respective medium, NA (0.1 µM, for 2 min) and ACh (10 µM, for 8 min) were added, and the medium was collected to measure the stimulated NO release. The fluorescence of the medium was measured at room temperature with a spectrofluorometer (LS50 Perkin Elmer Instruments, FL WINLAB Software) at an excitation wavelength of 492 nm and an emission wavelength of 515 nm. The results are expressed as the ratio between the ACh-induced release and the respective basal release.

In both aorta and mesenteric artery, blank measurements were also collected in the respective medium (without arterial segments) to subtract the background count values.

### 4.5. Detection of Superoxide Anion

The oxidative fluorescent probe, hydroethidine (HE, Sigma-Aldrich D7008), was used to evaluate superoxide anion levels in situ, as previously described [29,35,63,64]. Opened arterial segments for aorta and artery slices for mesenteric arteries were mounted on glass slides and imaged under a confocal microscope (Leica TCS ST2 DM IRE2) to detect nuclei (405 nm excitation and 410–475 nm emission for the DAPI dye) and oxidized HE (excitation 488 nm; emission 610 nm). The relative amount of superoxide formed was expressed as the ratio of the fluorescence emitted by HE to that emitted by DAPI with ImageJ analysis software (National Institutes of Health [NIH], RRID: SCR_003070).

### 4.6. Reagents

The drugs used (Sigma-Aldrich) were as follows: noradrenaline (NA) hydrochloride (A9512), acetylcholine (ACh) chloride (A6625), potassium chloride (P9541), sodium nitroprusside (SNP) (S0501), cromakalim (C1055), and CORM (288144). Stock solutions (10 mM) of cromakalim and CORM were prepared in absolute ethanol, and the final ethanol concentration in the tissue baths was less than 0.1% (*v*/*v*). NA was dissolved in a NaCl (0.9%)–ascorbic acid (0.01% w/v) solution, and other chemicals were dissolved in distilled water. The stock solutions were kept at −20 °C, and appropriate dilutions were prepared in KHS on the day of the experiment.

### 4.7. Statistical Analysis

The vasodilation induced by ACh, SNP, cromakalim, and CORM was expressed as a percentage of the initial contraction induced by NA. The vasoconstrictor response to KCl (75 mM) was expressed in mg. The values of NA-induced precontraction were expressed as percentage of the contraction induced by 75 mM KCl. Cumulative concentration response curves were built and EC_50_, E_max_, and the area under curves (AUC) values for all analyzed vasodilator responses were calculated. The results were compared by means of two-way analysis of variance (ANOVA) with Bonferroni’s multiple comparisons test. For NO release and superoxide anion production, statistical analysis was performed using Student’s *t*-test for unpaired experiments. The results are given as the means ± standard error of the mean (SEM). A *p* value of less than 0.05 was considered significant. The statistical analysis and the elaboration of the graphs were carried out using the statistical program GraphPad PRISM^®^ (Version 6.01).

## 5. Conclusions

The results show the antioxidant Enz-Spir-E improves vasorelaxant responses in vascular preparations from aged animals by increasing endothelial NO release/bioavailability, CO function, and K_ATP_ channel contribution to vasodilation, likely through superoxide anion scavenging. These data could support the potential use of this enzymatic extract for the prevention and treatment of cardiovascular disease. It would be worthwhile to conduct human studies using spirulina extracts as an alternative food supplement, always according to the clinical conditions.

## Figures and Tables

**Table 1:** Amino acid content of the spirulina extract obtained with Alcalase^®^.

	% *w*/*w*	µmol/g Extract
Asp	4.65 ± 1.05	404 ± 9
Thr	2.56 ± 0.62	222 ± 5
Ser	2.41 ± 0.79	209 ± 7
Glu	6.81 ± 1.23	591 ± 11
Pro	2.07 ± 0.12	180 ± 1
Gly	1.99 ± 0.31	173 ± 3
Ala	3.70 ± 0.43	322 ± 4
Cys	0.34 ± 0.04	30 ± 4
Val	2.80 ± 0.33	243 ± 3
Met	1.23 ± 0.15	107 ± 1
Ile	2.52 ± 0.49	219 ± 4
Leu	4.54 ± 0.74	394 ± 6
Tyr	1.71 ± 0.05	178 ± 0
Phe	2.40 ± 0.49	209 ± 4
His	0.66 ± 0.10	57 ± 1
Lys	2.28 ± 0.11	198 ± 1
Arg	2.31 ± 0.51	201 ± 4
**Total**	**45.0 ± 7.1**	**3907 ± 69**

**Table 2:** Peptide sequence, retention time (Rt, min.), calculated and experimental molecular mass (Da), and mark or score of the identified peptides in the hydrophilic extracts of spirulina by LC ESI-MS MS.

RT (min)	Peptide Sequence (Seq. ID:)	M_r_ (Da, Calc.)	M*_exp_ (Da)	MASCOT IONS SCORE **	Data Base
42.83	MKKIEAIIRPF (SEQ ID NO: 1)	1344.8	1344.7	46	gi|291569590, nitrogen regulatory protein P-II [*Arthrospira platensis* NIES-39]
51.60	LPPL (SEQ ID NO: 2)	438.3	438.3	N.P.	Not assignable to a concrete protein
52.48	ALAVGIGSIGPGLGQGQ (SEQ ID NO: 3)	1493.8	1493.7	90	gi|146186464, AtpH [*Arthrospira platensis* HN01]; gi|355333248, Chain B Microscopic Rotary Mechanism Of Ion Translocation In The Fo Complex Of Atp Synthases; gi|310689674, Chain E Microscopic Rotary Mechanism Of Ion Translocation In The Fo Complex Of Atp Synthases; gi|375325268, ATP synthase subunit C membrane-bound F0 sector; DCCD-binding [*Arthrospira* sp. PCC 8005]
53.79	TTAASVIAAAL (SEQ ID NO: 4)	987.6	987.4	40	gi|291566395, ATP synthase c chain [*Arthrospira platensis* NIES-39]
54.56	DFPGDDIPIVS (SEQ ID NO: 5)	1173.6	1173.5	40	gi|119213EFTU_ARTPT, RecName: full = elongation factor Tu; short = EF-Tu
54.90	LELL (SEQ ID NO: 6)	486.3	486.3	N.P.	Not assignable to a concrete protein
48.10	WKLLP (SEQ ID NO: 7)	655.4		de novo ***	
48.97	CHLLLSM (+15.99) (SEQ ID NO: 8)	831.4		*de novo* ***	

* A precision of ±0.1–0.2 Da was obtained, both for MS and MS/MS spectra. ** Statistical measure of confidence in the peptide identification. *** *De novo* analyses with a confidence level of 80.

**Table 3:** Effect of the Enz-Spir-E (0.1%*w*/*v*) incubation on vascular responses (E_max_, pEC_50_, and AUC) to the vasodilators acetylcholine (ACh), sodium nitroprusside (SNP), CO-releasing molecule (CORM), and cromakalim (CK) in aorta and mesenteric arteries from aged rats.

	E_max_		pEC_50_		AUC	
	Control	Enz-Spir-E	Control	Enz-Spir-E	Control	Enz-Spir-E
**Aorta**						
ACh	71.8 ± 5.5	96.8 ± 3.6 *	–7.2 ± 0.1	–8.5 ± 0.2 * (7/11)	(6/11) 69.7 ± 21.7	337.4 ± 30.0 *
SNP	97.5 ± 5.5	106.8 ± 4.9	–7.9 ± 0.1	–8.2 ± 0.2 (6/6)	(6/8) 291.4 ± 20.9	340.6 ± 20.9 *
CORM	44.0 ± 8.8	58.7 ± 19.7	–5.0 ± 0.3	–5.6 ± 1.2 (4/4)	(5/5) 48.2 ± 7.8	90.4 ± 23.2 *
CK	93.0 ± 3.1	100.0 ± 5.9	–6.6 ± 0.4	–6.0 ± 0.3 * (5/6)	(5/7) 211.3 ± 24.1	185.8 ± 41.3
**Mesenteric Artery**						
ACh	99.7 ± 6	120.3 ± 5.3 *	–7.7 ± 0.1	–10.4 ± 0.2 * (7/11)	(6/7) 297.1 ± 28.4	633.4 ± 44.3 *
SNP	108.4 ± 4.9	120.2 ± 8.7	–8.0 ± 0.2	–8.0 ± 0.5 (6/8)	(4/4) 345.0 ± 28.1	446.4 ± 50.5 *
CORM	45.3 ± 5.9	44.7 ± 6.8	–4.7 ± 0.5	–5.3 ± 0.4 * (5/6)	(4/4) 49.1 ± 10.3	60.3 ± 10.3 *
CK	101.5 ± 3.2	121.7 ± 5.3	–6.5 ± 0.1	–6.1 ± 0.3 (4/4)	(5,6) 194.8 ± 19.8	338.0 ± 37.8 *

Values are expressed as mean ± SEM. Emax: maximal vasodilator response; pEC_50_: logarithm of effective concentration 50; AUC: area under curve. Numbers in parentheses indicate the number of animals and arterial segments. * *p* < 0.05 with respect to control condition.

## Data Availability

All the data are included within the article.

## Supplementary Materials

The following supporting information can be downloaded at: https://www.mdpi.com/article/10.3390/md23100395/s1, Individual experimental data points for Figure 1, Figure 2, Figure 3 and Figure 4 are included in the Supplementary information file.

## Abbreviations

The following abbreviations are used in this manuscript:

ACh	Acetylcholine
CO	Carbon monoxide
CORM	Carbon monoxide releasing molecules
CVD	Cardiovascular diseases
DAF-2	4,5-diaminofluorescein
Enz-Spir-E	Enzymatic Spirulina Extract
HE	Hydroethidine
K_ATP_	ATP dependent potassium channel
KHS	Krebs–Henseleit solution
NO	Nitric oxide
ROS	Reactive oxygen species
SNP	Sodium nitroprusside
